# Targeting CDK4/6 in breast cancer

**DOI:** 10.1038/s12276-025-01395-3

**Published:** 2025-02-10

**Authors:** Anusha Shanabag, Jessica Armand, Eugene Son, Hee Won Yang

**Affiliations:** 1 Department of Pathology and Cell Biology, Columbia University, New York, NY USA; 2 Herbert Irving Comprehensive Cancer Center, Columbia University, New York, NY USA

**Keywords:** Breast cancer, Checkpoints

## Abstract

Dysregulation of the cell cycle machinery, particularly the overactivation of cyclin-dependent kinases 4 and 6 (CDK4/6), is a hallmark of breast cancer pathogenesis. The introduction of CDK4/6 inhibitors has transformed the treatment landscape for hormone receptor-positive breast cancer by effectively targeting abnormal cell cycle progression. However, despite their initial clinical success, drug resistance remains a significant challenge, with no reliable biomarkers available to predict treatment response or guide strategies for managing resistant populations. Consequently, numerous studies have sought to investigate the mechanisms driving resistance to optimize the therapeutic use of CDK4/6 inhibitors and improve patient outcomes. Here we examine the molecular mechanisms regulating the cell cycle, current clinical applications of CDK4/6 inhibitors in breast cancer, and key mechanisms contributing to drug resistance. Furthermore, we discuss emerging predictive biomarkers and highlight potential directions for overcoming resistance and enhancing therapeutic efficacy.

## Targeting cell cycle dysregulation to combat breast cancer

CDK4/6 inhibitors have revolutionized the treatment of hormone receptor-positive breast cancer by targeting abnormal cell growth. However, most patients eventually encounter drug resistance, and predicting responses remains a challenge. This Review delves into the mechanisms behind CDK4/6 inhibitor resistance and explores potential strategies to overcome it. The authors provide a comprehensive overview of the cell cycle and the role of CDK4/6 inhibitors, highlighting both genetic and nongenetic factors that drive resistance. Key insights reveal that mutations and alterations in signaling pathways significantly contribute to drug resistance, offering avenues for novel therapeutic targets. Moreover, the Review emphasizes the importance of biomarkers to better predict treatment outcomes. Understanding these resistance mechanisms is pivotal for developing advanced strategies to enhance therapy effectiveness and improve patient prognosis.

This summary was initially drafted using artificial intelligence, then revised and fact-checked by the author.

## Introduction

Breast cancer is one of the most common cancers worldwide and a leading cause of cancer-related mortality among women^1^. Despite notable progress in early detection and treatment, resistance to conventional therapies remains a major challenge, underscoring the need for innovative therapeutic approaches. Targeting the cell cycle—a fundamental process that controls cell proliferation—presents a promising strategy in cancer treatment^2^. Dysregulation of the cell cycle is a hallmark of cancer, driving uncontrolled cell division and tumor growth^3^.

The cell cycle comprises a series of tightly regulated phases, ensuring the orderly progression of cells through growth, DNA replication and division^4^. Critical regulators of this process include cyclins, cyclin-dependent kinases (CDKs), CDK-inhibitor proteins and the retinoblastoma protein (Rb), which cooperatively control the transition between different cell cycle phases^4,5^. Alterations in these regulatory molecules can lead to aberrant cell proliferation in cancer. Overexpression of cyclin D is observed in 50–60% of breast cancer cases, indicating CDK4/6 overactivation^6^. Furthermore, cyclin E overexpression, CDK4/6 amplification and loss-of-function (LOF) mutations in tumor suppressor genes, such as p53, are frequently found in breast cancer and induce abnormal cell cycle progression^7^.

Breast cancer is classified on the basis of the expression of the following receptors: hormone receptors (HR: estrogen and progesterone receptors) and human epidermal growth factor receptor 2 (HER2). CDK4/6 inhibitors have emerged as promising therapeutic agents in HR-positive (HR^+^)/HER2-negative (HER2^−^) breast cancer. These inhibitors selectively target CDK4/6 to prevent Rb phosphorylation in the G1 phase, thereby blocking the G1/S transition^8^. Clinical trials have demonstrated that the addition of CDK4/6 inhibitors to endocrine therapy significantly improves progression-free survival (PFS) in patients with metastatic HR^+^/HER2^−^ breast cancer^9–12^. However, despite their success, developing resistance to CDK4/6 inhibitors poses a significant challenge, promoting research into understanding resistance mechanisms and identifying new therapeutic targets.

Here, we discuss the molecular mechanisms underlying cell cycle regulation and resistance to CDK4/6 inhibitors in breast cancer. We also explore potential predictive biomarkers and therapeutic strategies to overcome drug resistance, providing an overview of current directions for targeting the cell cycle in breast cancer therapy.

## Mechanisms of cell cycle entry

Entry into the cell cycle is tightly regulated to ensure cells advance to the S phase when all requisite conditions are satisfied^13^. Rb binds and sequesters E2F transcription factors during quiescence to prevent their activity and premature cell cycle entry^14^. In the canonical pathway, mitogenic stimulation induces cyclin D expression, which binds and activates CDK4/6, forming the active cyclin D–CDK4/6 complex^15,16^. Active CDK4/6 phosphorylates Rb, leading to the effective release of E2F transcription factors. The Rb-related pocket proteins Rbl1 (p107) and Rbl2 (p130), which recruit chromatin repressor complexes, are also substrates of CDK4/6^17^. Released E2Fs initiate the transcription of genes involved in S-phase entry, including the CDK2 activators cyclins E and A^17,18^. The activation of CDK2 further phosphorylates Rb and various substrates necessary for DNA synthesis and replication, thereby driving S-phase entry^19,20^. Moreover, E2Fs regulate the expression of genes involved in nucleotide biosynthesis, such as *DHFR*, *RRM1* and *RRM2*, and mitotic progression, including *PLK1*, *BUB1* and *MAD2*^17,18^.

In addition to cyclin binding, the full activation of CDK4/6 requires the phosphorylation of its T-loop by CDK-activating kinase (CAK)^21–23^. CAKs consist of CDK7, cyclin H and the assembly factor MAT1. The phosphorylation of the T-loop displaces it from the active site, allowing the formation of a functional cyclin–CDK–substrate complex. CAK also phosphorylates RNA polymerase II to regulate mRNA transcription, linking cell cycle regulation with transcriptional control^24^.

Several regulatory mechanisms suppress CDK4/6 activity under various conditions, such as contact inhibition, activation of the p53/p21 pathway, limited availability of mitogens, and expression of CDK inhibitor proteins^25^. The INK4 family of CDK4/6 inhibitors (p16, p15, p18 and p19) bind to the catalytic pocket of CDK4/6, competing with cyclin D and blocking CDK4/6 kinase activity^26^. Cyclin D is inherently unstable and undergoes phosphorylation at the end of the G1 phase, which enables its export to the cytoplasm via CRM1^27^. In addition, recent genome-wide screening studies have identified autophagy and beclin 1 regulator 1 (AMBRA1) as an E3 ligase adaptor that regulates cyclin D levels through ubiquitylation and proteasomal degradation^28–30^. Cip/Kip family proteins (p21, p27 and p57) bind and inhibit active cyclin–CDK complexes, suppressing cell cycle progression^31^. However, previous studies have shown that these proteins can stabilize cyclin D–CDK4/6 complexes and facilitate their nuclear translocation^26,32,33^. Whereas Cip/Kip family proteins typically inactivate CDK4/6^34–36^, phosphorylated p27 has been shown to activate CDK4/6 by dislodging itself from the CDK active site^36,37^. Nevertheless, other studies have demonstrated CDK4/6 activation in mouse embryonic fibroblasts lacking p21 and p27, as well as in nontransformed cell lines lacking p21, p27 and p57^38,39^, indicating the need for further investigation into the biological significance of p27 phosphorylation.

While CDK4/6 plays a central role in cell cycle entry through Rb phosphorylation, several studies have shown that cells can progress through the cell cycle in the absence of CDK4/6 activity. Early reports demonstrated cell proliferation in mice lacking all cyclin D1–3 isoforms or CDK4/6^40,41^ and the initiation of DNA replication without CDK4/6 activity and Rb phosphorylation^42^. One possible mechanism is the dilution of the Rb concentration due to cell growth, which contributes to CDK4/6-independent Rb inactivation^43^. In addition, recent findings have indicated that nonphosphorylated Rb is intrinsically unstable, leading to reduced protein levels^44^. However, this passive Rb inactivation is insufficient for full E2F activation, necessitating a secondary amplification step. This amplification is driven primarily by the global transcriptional enhancer c-Myc, which is upregulated by mitogenic and hormone signaling^45,46^. Importantly, while c-Myc overexpression induces cyclin D expression to activate CDK4/6, it amplifies E2F activity following Rb reduction under CDK4/6 inhibition^44^. Other studies have also shown that c-Myc overexpression facilitates CDK2 activation, promoting cell cycle entry through a noncanonical pathway^42,47^. Several E3 ubiquitin ligases, including NRBE3, SETDB1, β-TRCP1 and UBR5, have been identified as mediators of Rb degradation^48–51^. By contrast, other studies failed to detect ubiquitination-dependent Rb degradation^52–54^; instead, they suggested that MDM2^52^ and TTC39B^53^ promote Rb degradation through ubiquitination-independent, proteasome-driven mechanisms.

This comprehensive understanding of both the canonical and noncanonical pathways of cell cycle entry highlights the complex regulatory networks involved in cell proliferation, suggesting potential therapeutic targets and strategies for treating cancers characterized by cell cycle dysregulation.

## Role of CDK4/6 inhibitors in breast cancer therapy

Over two decades ago, the first generation of CDK inhibitors, often called pan-CDK inhibitors, was developed as potential cancer therapeutics. However, the lack of selectivity led to significant toxicity and failure in clinical trials^55^. A breakthrough came with the development of selective CDK4/6 inhibitors, which have demonstrated efficacy in human luminal/estrogen receptor-positive cancer cells, suggesting their potential utility in breast cancer treatment^56^. Unlike first-generation inhibitors, these small molecules exhibit increased selectivity toward CDK4/6 and reduced toxicity^57^. CDK4/6 inhibition blocks cell cycle progression in the G1 phase and induces cellular senescence. HR^+^/HER2^−^ breast cancer, which typically retains Rb function and activates the cyclin D–CDK4/6 pathway via estrogen signaling, has the highest sensitivity to CDK4/6 inhibition^55^. The promising efficacy of these inhibitors led to a series of clinical trials and subsequent Food and Drug Administration (FDA) approval of three CDK4/6 inhibitors—palbociclib (Ibrance), ribociclib (Kisqali) and abemaciclib (Verzenio)—for the treatment of HR^+^/HER2^−^ metastatic breast cancer in combination with endocrine therapy. Several clinical trials, including MONARCH-3, PALOMA-1, PALOMA-2, MONALEESA-2 and MONALEESA-7, have evaluated the efficacy of these inhibitors in combination with endocrine therapy as a first-line treatment, demonstrating significant improvements in PFS compared with endocrine monotherapy^58–62^. Unlike palbociclib and ribociclib, abemaciclib has been approved as monotherapy owing to its additional off-target effects^63^. These promising results led to the combination of CDK4/6 inhibitors and endocrine therapy as standard therapy for HR^+^/HER2^−^ metastatic breast cancer.

Recently, the China FDA approved dalpiciclib (Herngri) for the treatment of HR^+^/HER2^−^ metastatic breast cancer^64^. The DAWNA-2 trial, which evaluated the efficacy of dalpiciclib in combination with endocrine therapy as a first-line treatment, reported a median PFS exceeding 30 months and a 49% reduction in the risk of disease progression^65^. Compared with endocrine monotherapy, the PALOMA-3, MONARCH-2 and MONALEESA-3 trials assessed CDK4/6 inhibitors in combination with endocrine therapy in endocrine therapy-resistant populations and demonstrated significant improvements in median PFS and objective response rates^10,12,59^. However, the impact on overall survival (OS) varied across these trials, with MONARCH-2 and MONALEESA-3 showing significant improvement in OS, whereas PALOMA-3 displayed a trend toward improvement in OS that did not reach statistical significance^66^.

The clinical success of CDK4/6 inhibitors in HR^+^/HER2^−^ metastatic breast cancer has led to a significant expansion in their application, with over 100 clinical trials investigating CDK4/6 inhibitors in combination with chemotherapy, immunotherapy or other targeted therapies across different breast cancer subtypes and other tumor types (Table 1)^67^. For example, in HR^+^/HER2^+^ breast cancer, the HER2 signaling pathway enhances CDK4/6 activity, making CDK4/6 an ideal therapeutic target^66^. Six ongoing trials are exploring approved CDK4/6 inhibitors combined with endocrine therapy and/or anti-HER2 therapy for HR^+^/HER2^+^ metastatic breast cancer, with PFS or OS as the primary endpoint (NCT03304080, NCT02947685, NCT05969184, NCT03772353, NCT05800756 and NCT04334330). For triple-negative breast cancer (TNBC), four ongoing clinical trials are evaluating FDA-approved CDK4/6 inhibitors as monotherapies (NCT03979508) in combination with anti-androgen receptor (AR) therapy in AR-positive (AR^+^) populations (NCT02605486 and NCT03090165) or with a histone deacetylase (HDAC) inhibitor (NCT04315233). However, owing to the heterogeneous nature of TNBC, findings from different in vitro studies have been controversial, with some reporting insensitivity to CDK4/6 inhibition. By contrast, others, particularly in AR^+^ cell lines, have shown sensitivity^68^. In early-stage HR^+^ breast cancer, CDK4/6 inhibitors are being investigated for their potential use. Several translational studies using tumor biopsies have demonstrated that neoadjuvant endocrine therapy combined with CDK4/6 inhibition can reduce Ki67 expression and induce cellular senescence^8,55^. However, the clinical data have been inconsistent. The PENELOPE-B^69^ and PALLAS^70^ trials did not show significant improvements in PFS. By contrast, the monarchE trial reported significant improvement in PFS, leading to the approval of adjuvant abemaciclib therapy for patients with high-risk early HR^+^ breast cancer (Ki67 index >20%)^71^. In addition, four other clinical trials are evaluating PFS in early HR^+^/HER^−^ patient populations receiving adjuvant endocrine therapy in combination with CDK4/6 inhibitors (NCT02513394, NCT02764541, NCT04293393 and NCT06341894). Notably, the NATALEE trial will assess PFS in 5,000 patients treated with ribociclib over 3 years, representing the most extended treatment duration among these trials (NCT03701334).

**Table 1:** Ongoing clinical trials of CDK4/6 inhibitors in patients with HR^+^ breast cancer, HER2^+^ breast cancer or TNBC.

Addition	CDK4/6 inhibitor	Phase	Tumor type	Start	Estimated completion	Trial
**HR**^**+**^ **breast cancer**						
Endocrine therapy + locoregional treatment	Palbociclib	N/A	Treatment naive, HR^+^/HER2^−^ metastatic	2019-10-23	2027-10-23	NCT03870919 (PALATINE)
Endocrine therapy	Palbociclib	II	Slowly proliferating, HR^+^ breast cancer	2015-10	2029-02	NCT02592083 (PREDIX LumA)
Endocrine therapy	Palbociclib	II	HR^+^ metastatic breast cancer	2015-02	2031-12-31	NCT02603679 (PREDIX LumB)
Endocrine therapy	Palbociclib, Ribociclib	III	HR^+^ metastatic breast cancer	2017-11-09	2025-12	NCT03425838 (SONIA)
Endocrine therapy	Palbociclib	III	HR^+^ metastatic breast cancer, treatment naive	2015-03-23	2025-02-28	NCT02297438 (PALOMA-4)
Endocrine therapy	Palbociclib	II	HR^+^/HER2^−^ metastatic breast cancer	2016-12-07	2026-06-30	NCT02942355
Endocrine therapy	Palbociclib	III	HR^+^/HER2^−^ advanced breast cancer	2018-02-09	2025-09	NCT03423199 (PATHWAY)
Endocrine therapy	Palbociclib	II	Stage II/III HR^+^/HER2^−^ breast cancer	2013-04-10	2026-08-24	NCT01723774
Endocrine therapy	Palbociclib	II	HR^+^/HER2^−^ metastatic breast cancer	2016-10-25	2025-07	NCT02738866
Endocrine therapy	Palbociclib	III	HR^+^/HER2^−^ early breast cancer	2015-08	2025-09	NCT02513394 (PALLAS)
Endocrine therapy	Palbociclib	II	Unresectable stage I HR^+^/HER2^−^ breast cancer	2017-02-16	2024-12-31	NCT02760030
Endocrine therapy	Palbociclib	III	HR^+^/HER2^−^ metastatic breast cancer	2017-03-22	2025-06-30	NCT03079011
Endocrine therapy	Palbociclib	II	HR^+^ early-stage breast cancer	2016-05-24	2031-04	NCT02764541 (PELOPS)
Endocrine therapy	Palbociclib	III	HR^+^/HER2^−^ metastatic or locally advanced breast cancer	2020-10-09	2028-03-25	NCT04546009
Endocrine therapy	Palbociclib	I/II	HR^+^ advanced breast cancer	2017-09-20	2027-12-29	NCT03284957
Endocrine therapy	Palbociclib	I	HR^+^/HER2^−^ metastatic or advanced breast cancer	2017-11-27	2025-06-30	NCT03332797
Endocrine therapy	Palbociclib	III	HR^+^/HER2^−^ breast cancer, resected isolated locoregional recurrence	2019-08-27	2026-11-01	NCT03820830 (POLAR)
Endocrine therapy	Ribociclib	II	HR^+^/HER2^−^ advanced breast cancer	2018-08-29	2025-06-16	NCT03671330
Endocrine therapy	Ribociclib	II	HR^+^/HER2^−^ advanced breast cancer	2018-12-27	2025-11-27	NCT03944434 (FACILE)
Endocrine therapy	Abemaciclib	II	HR^+^/HER2^−^ advanced breast cancer	2020-01-07	2023-12	NCT04227327
Endocrine therapy	Abemaciclib	II	HR^+^/HER2^−^ advanced breast cancer	2020-09-14	2024-12-30	NCT04352777
Endocrine therapy	Abemaciclib	II	HR^+^/HER2^−^ early BREAST CANcer	2020-10-02	2033-02-28	NCT04293393
Endocrine therapy	Abemaciclib	I	HR^+^/HER2^−^ metastatic breast cancer	2014-03-10	2024-12	NCT02057133
Endocrine therapy	Abemaciclib	III	HR^+^/HER2^−^ metastatic breast cancer	2020-06-11	2028-06	NCT04158362
Endocrine therapy	Dalpiciclib	II	HR^+^/HER2^−^ early breast cancer	2023-11-17	2029-06	NCT06341894
Endocrine therapy	Ribociclib	III	HR^+^/HER2^−^ early breast cancer	2018-12-07	2026-05-29	NCT03701334 (NATALEE)
**HER2**^**+**^ **breast cancer**						
Anti-HER2	Palbociclib	I/II	HR^+^/HER2^+^ metastatic breast cancer	2017-12-20	2025-12-31	NCT03304080
Anti-HER2	Palbociclib	III	HR^+^/HER2^+^ metastatic breast cancer	2017-06-21	2026-07-31	NCT02947685
Endocrine therapy + anti-HER2	Palbociclib	II	HR^+^/HER2^+^ metastatic breast cancer	2021-12-25	2024-12-25	NCT05969184
Endocrine therapy + anti-HER2	Dalpiciclib	I/II	HR^+^/HER2^+^ metastatic breast cancer	2019-05-12	2025-12-10	NCT03772353
Endocrine therapy + anti-HER2	Dalpiciclib	II	Stage II–III HR^+^/HER2^+^ breast cancer	2022-08-04	2025-07-30	NCT05800756
Endocrine therapy + anti-HER2	Palbociclib	II	HR^+^/HER2^+^ breast cancer with brain metastases	2020-12-04	2024-12-31	NCT04334330
**TNBC**						
AR inhibitor	Palbociclib	I/II	AR^+^, HR^−^/HER2^−^ metastatic breast cancer	2015-11-11	2025-11	NCT02605486
AR inhibitor	Ribociclib	I/II	AR^+^, HR^−^/HER2^−^ advanced breast cancer	2018-05-07	2025-09	NCT03090165
Not applicable	Abemaciclib	II	AR^+^, HR^−^/HER2^−^ stage I–III breast cancer	2020-01-10	2025-07-31	NCT03979508
HDAC inhibitor	Ribociclib	I	HR^−^/HER2^−^ metastatic breast cancer	2021-05-03	2026-08	NCT04315233

The expanding role of CDK4/6 inhibitors in breast cancer therapy underscores their importance in both metastatic and early-stage disease, with ongoing research focused on refining their application across breast cancer subtypes.

## Mechanisms of resistance to CDK4/6 inhibitors

Despite the initial success of CDK4/6 inhibitors in treating HR^+^/HER2^−^ breast cancer, most patients eventually develop drug resistance, limiting the long-term efficacy of this treatment^72,73^. To address this, extensive research has focused on elucidating the mechanisms driving resistance. Approximately 30% of patients with HR^+^/HER2^−^ breast cancer who develop resistance to CDK4/6 inhibitors acquire new genetic mutations^11^. As Rb is the primary substrate of CDK4/6, LOF mutations in *Rb* represent the most well-documented mechanisms in preclinical models^2,67^. Breast cancer cells harboring *Rb* LOF mutations are unresponsive to CDK4/6 inhibitors, but this primary resistance can be reversed by reintroducing wild-type Rb^54^. However, *Rb* LOF mutations are relatively rare in HR^+^/HER2^−^ breast cancer, occurring in only 4.7% of cases^11,74^. Another notable mutation involves LOF mutations in *FAT1*, which are found in approximately 6% of HR^+^/HER2^−^ metastatic breast cancers^75^. *FAT1* mutations drive CDK6 overexpression via the Hippo pathway, leading to the formation of CDK6–INK4 complexes that are resistant to CDK4/6 inhibitors^75,76^. CDK6 amplification and the formation of distinct CDK6 complexes have also been consistently implicated in resistance^77,78^.

**Fig. 1:**
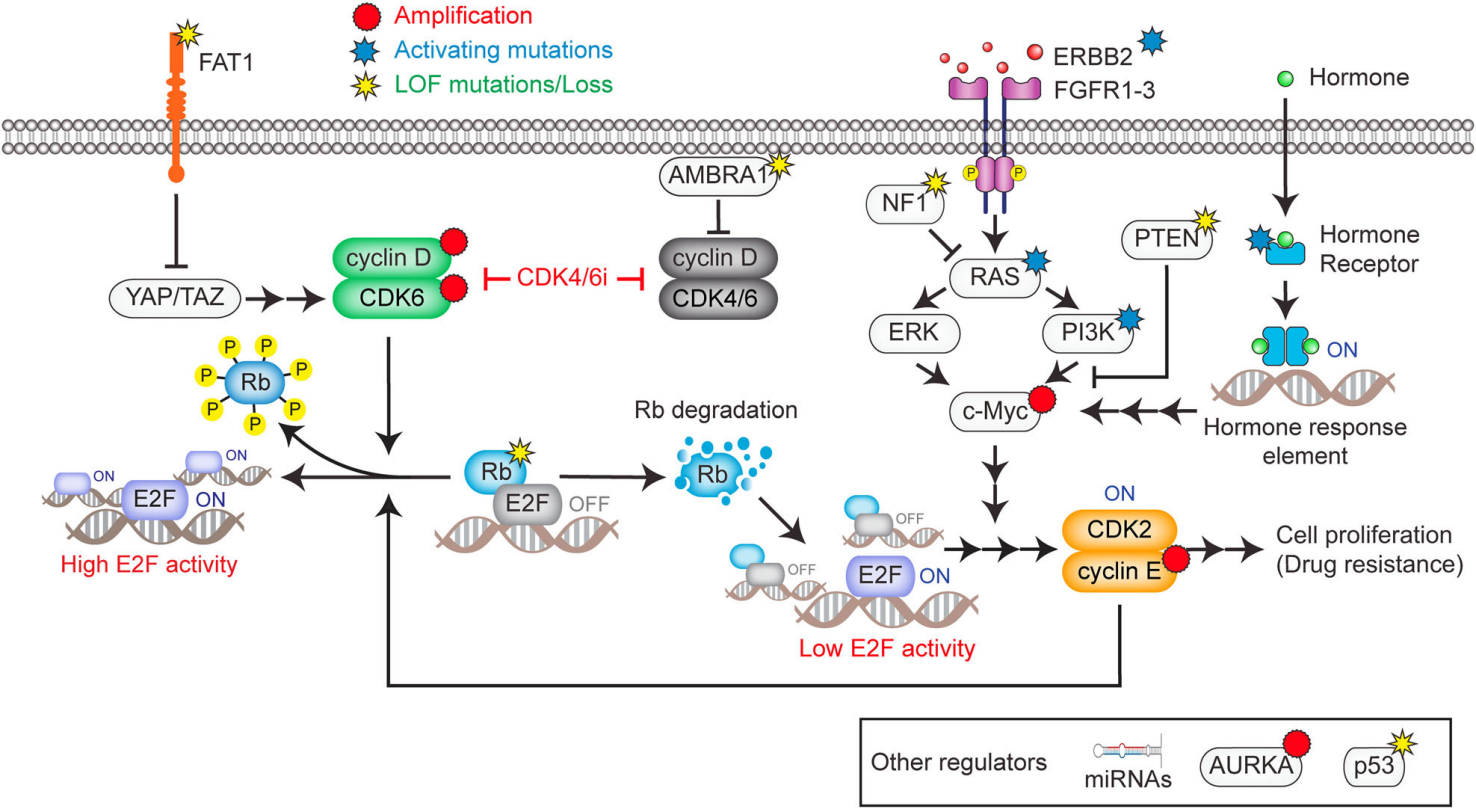
Resistance mechanisms to CDK4/6 inhibitors. This schematic illustrates the key genetic and nongenetic mechanisms contributing to resistance against CDK4/6 inhibitors in HR^+^/HER2^−^ breast cancer. In the canonical pathway, mitogenic and hormone-signaling pathways activate the cyclin D–CDK4/6 complex, which phosphorylates Rb, effectively releasing E2F transcription factors and promoting cell cycle entry. LOF mutations in *Rb* represent the most well-documented primary resistance mechanism to CDK4/6 inhibitors. Another genetic mechanism involves *FAT1* LOF mutations, which activate the Hippo pathway, leading to CDK6 overexpression and the formation of CDK6‒INK4 complexes resistant to CDK4/6 inhibitors. Mutations in mitogenic and hormone-signaling pathways, such as *ERBB2*, *FGFR1-3*, *PIK3CA*, *NF1* and *ESR1*, which can increase *c-Myc* expression, are frequently observed in CDK4/6 inhibitor-resistant tumors. Nongenetic mechanisms include Rb degradation, bypassing CDK4/6 inhibition to alternatively enter the cell cycle. However, this noncanonical pathway for Rb inactivation is incomplete, necessitating E2F amplification by *c-Myc*, which has been shown to inversely correlate with the outcomes of CDK4/6 inhibitor therapies. The loss of AMBRA1 stabilizes cyclin D and enables its interaction with CDK2, driving resistance. In addition, amplification of *AURKA* and *CCNE1/2* upregulates CDK2 activity, allowing tumor cells to bypass the dependency on CDK4/6. Alterations in specific miRNAs also contribute to CDK4/6 inhibitor resistance. This model emphasizes the multifaceted nature of resistance mechanisms, highlighting the roles of genetic mutations, signaling pathway alterations and transcriptional regulation.

The mitogenic and hormone-signaling pathways induce cyclin D expression to activate CDK4/6, which is crucial in determining sensitivity to CDK4/6 inhibitors (Fig. 1). Tumors exhibiting increased FGFR2 or ERBB3 expression are associated with improved PFS when treated with CDK4/6 inhibitors^79^. However, numerous studies have demonstrated the association of mitogenic and hormone signaling with resistance to CDK4/6 inhibitors^11,74,80–83^. For example, amplification of the FGFR signaling pathway and PTEN loss are frequently observed in resistant tumors^80,84^, and alterations in *KRAS* have been linked to shorter PFS in HR^+^/HER2^−^ breast cancer^85^. Most driver mutations that promote resistance are found in mitogenic and hormone-signaling genes, such as *PIK3CA*, *ESR1*, *ERBB2*, *NF1* and *FGFR1-3*^11,74,80–82^. Nevertheless, breast cancer can still respond to CDK4/6 inhibitors irrespective of *ESR1* mutation status^86^, and *PIK3CA* alterations are present in both sensitive and resistant tumors^74^. These findings suggest that these genetic alterations may facilitate resistance while allowing an initial response to CDK4/6 inhibitors.

Approximately 70% of patients with HR^+^/HER2^−^ breast cancer who initially respond to CDK4/6 inhibitors develop resistance without acquiring new somatic mutations^11^, highlighting the role of nongenetic mechanisms. A previous study revealed the formation of a noncanonical cyclin D–CDK2 complex in HR^+^ breast cancer cells, leading to resistance through alternative CDK2 activation^87^. Loss of *AMBRA1* has been shown to promote cyclin D overexpression, facilitating its interaction with CDK2^28,30^. In addition, recent findings indicate that nonphosphorylated Rb is inherently unstable and undergoes proteolytic degradation^54,88^. While this Rb degradation can initiate E2F activation independently of CDK4/6, it provides only partial and low-level activation of E2F. The global transcription amplifier c-Myc plays a critical role in the development of CDK4/6 inhibitor resistance by increasing this low E2F activity. Therefore, in pretreatment samples, c-Myc expression levels, rather than Rb levels, inversely correlate with the therapeutic efficacy of combined CDK4/6 inhibitors and endocrine therapy as a first-line treatment in HR^+^/HER2^−^ breast cancer. Consistently, pre- and post-treatment circulating tumor DNA analysis revealed increased c-Myc amplification in patients treated with abemaciclib compared with those treated with placebo, suggesting that c-Myc amplification may serve as a potential resistance mechanism^89^. This finding aligns with the frequent mutations found in mitogenic and hormone-signaling pathways that upregulate c-Myc expression in tumors resistant to CDK4/6 inhibitors. This may also account for *PIK3CA* mutations in both sensitive and resistant tumors^34^, as well as the efficacy of CDK4/6 inhibitors regardless of *ESR1* mutation status^36^. Because hormone-signaling pathways upregulate c-Myc^90^, this may explain the synergistic effects observed between CDK4/6 inhibitors and endocrine therapy. Moreover, additional studies have highlighted the role of c-Myc in metabolic adaptation^91^ and CDK6 overexpression^92^, contributing to resistance.

The amplification of *Aurora kinase A* (*AURKA*), which regulates the G2/M transition, has been observed in 27% of HR^+^ breast tumors resistant to CDK4/6 inhibitors^74^. By contrast, tumors sensitive to CDK4/6 inhibitors retain regular AURKA expression. Amplifications of *CCNE1* and *CCNE2* (cyclin E1 and E2) are also frequently associated with resistance, as cyclin E upregulation enhances CDK2 activity, bypassing the need for CDK4/6 activity^74,93,94^. In one study, *CCNE1* amplification was observed in HR^+^/HER2^−^ cell lines with acquired resistance to palbociclib^87^. Inhibition of cyclin E1 or CDK2 restored sensitivity to CDK4/6 inhibitors, suggesting that cyclin E1 drives CDK2-dependent cell cycle progression in resistant cells.

MicroRNA (miRNA), noncoding RNA molecules, have also been implicated in mediating resistance to CDK4/6 inhibitors^95^. For example, the upregulation of miRNA-432-5p in resistant HR^+^/HER2^−^ cells suppresses SMAD4 in the TGF-β pathway, leading to CDK6 upregulation and CDK4/6-inhibitor resistance^96^. Similarly, miRNA-29b-3p also regulates CDK6 expression and resistance^92^. miRNA-223 has been suggested as a potential marker of resistance, with its loss correlating with reduced OS in patients with breast cancer^97^. Other miRNAs, including miRNA-126, miRNA-326 and miRNA-3613-3p, have also been associated with sensitivity to CDK4/6 inhibitors in breast cancer cell lines^95,98^.

Emerging evidence indicates that CDK4/6 inhibition significantly alters the epigenetic landscape. A previous study showed that CDK4/6 inhibition upregulates the expression and activation of AP-1, a transcription factor that enhances the accessibility of regulatory enhancers^99^. These enhancers govern critical processes such as cellular differentiation, evasion of apoptosis, and long terminal repeats that may influence tumor immunogenicity. Another study revealed that the nonreceptor tyrosine kinase ACK1 is upregulated in tumors treated with CDK4/6 inhibitors^100^. This study indicates that ACK1 contributes to resistance by depositing epigenetic marks at key cell cycle regulator genes, including cyclin B1, B2 and CDC20. In TNBC models, BET inhibitors, which target regulators of transcriptional elements, have shown synergy with CDK4/6 inhibitors^101^. This combination disrupts mitosis, induces chromosomal instability and ultimately drives cellular senescence, suggesting a potential strategy to overcome resistance.

## Potential predictive biomarkers of response to CDK4/6 inhibitors

The therapeutic efficacy of CDK4/6 inhibitors varies among patients with HR^+^/HER^−^ breast cancer^102,103^, underscoring the need to identify reliable biomarkers that can predict the response to these treatments.

### LOF mutations in *Rb* genes and Rb mRNA and protein levels

LOF mutations in the *Rb* gene represent one of the most prominent biomarkers for predicting primary resistance to CDK4/6 inhibitors^79,104^. A recent study identified germline *BRCA2*-mutant breast cancer as having significant enrichment of *Rb* LOF mutations, leading to poor PFS with CDK4/6 inhibitors^105^. However, in HR^+^/HER2^−^ breast cancer, complete loss of Rb function is clinically rare, occurring in approximately 4.7% of cases^11,74^. In contrast to *Rb* LOF mutations, variations in Rb mRNA and protein expression among patients with intact Rb function do not reliably predict the response to CDK4/6 inhibitors^104,106–108^. These findings suggest that, while *Rb* LOF mutations are a clear driver of primary resistance, Rb mRNA or protein levels may not be sufficient to influence therapeutic outcomes in patients with functional Rb.

### Cyclin D mRNA and protein levels

Given the role of cyclin D in activating CDK4/6, preclinical studies of breast cancer cell lines have revealed elevated levels of cyclin D proteins in cells sensitive to palbociclib^109–111^. These findings sparked interest in whether baseline cyclin D levels could predict patient response to CDK4/6 inhibitors. However, clinical data have shown that baseline cyclin D levels do not significantly correlate with patient response to CDK4/6 inhibitors^106,108,112^. Consistent with these clinical observations, basic research indicates that the ratio of cyclin D to Cip/Kip family members is a more reliable predictor of CDK4/6 activation than cyclin D levels alone^34–36^. Stabilizing cyclin D by Cip/Kip proteins can result in even higher cyclin D levels in quiescent cells than in proliferating cells, complicating the interpretation of cyclin D expression levels as a biomarker of CDK4/6 activation^34^.

### LOF mutation in *FAT1* gene

Genomic analysis of baseline HR^+^/HER^−^ breast cancer biopsy samples revealed LOF mutations in the *FAT1* gene as a significant predictor of early progression on CDK4/6 inhibitors. These mutations are found in approximately 6% of metastatic and 2% of primary tumor samples^75^.

### LOF mutations in *TP53* (p53) gene

Genomic analyses showed that LOF mutations in the *TP53* gene are inversely correlated with PFS in patients treated with CDK4/6 inhibitors^89,108,113^. A recent study further demonstrated that *TP53* LOF mutations can influence the long-term response to CDK4/6 inhibitors in breast cancer^114^. Despite this, patients with *TP53* LOF mutations still responded to CDK4/6 inhibitor therapy and exhibited worse PFS, even in the control groups. These findings suggest that, although *TP53* LOF mutations are associated with more aggressive tumor growth and reduced OS, they do not necessarily confer resistance to CDK4/6 inhibitors. Instead, *TP53*-mutated tumors are more likely to demonstrate rapid progression on CDK4/6 inhibitors.

### Mitogenic and hormone signaling and c-Myc

Genetic mutations in key components of mitogenic and hormone-signaling pathways, such as *PIK3CA*, *ESR1*, *FGFR1-3*, *KRAS* and *PTEN*, are frequently identified in tumors resistant to CDK4/6 inhibitors^11,74,80–84^. Furthermore, baseline analysis of HR^+^/HER2^−^ breast cancer samples revealed that phosphorylated AKT, which is indicative of PI3K pathway activation, along with c-Myc gene amplification and elevated c-Myc protein levels, was inversely correlated with PFS in patients receiving CDK4/6 inhibitors combined with endocrine therapy^54,115^. These findings suggest that both c-Myc and its regulatory pathways could serve as predictive biomarkers for assessing the efficacy of CDK4/6 inhibitors in breast cancer therapy.

### *CCNE1* (cyclin E1) amplification

The amplification of cyclin E1 is a known mechanism of CDK4/6 inhibitor resistance involving the activation of the cyclin E-CDK2 complex to promote S phase entry and bypass CDK4/6 activation. In breast tumor biopsies, *CCNE1* overexpression was shown to significantly correlate with resistance to palbociclib^74^, whereas lower *CCNE1* mRNA levels were associated with improved therapeutic response in HR^+^ breast cancer^107,116^. In the PALOMA-3 and NeoPalAna trials, high *CCNE1* expression was associated with lower PFS and relative resistance to palbociclib^107,117^. Furthermore, selective CDK2 inhibitor treatment in *CCNE1*-amplified tumors resistant to CDK4/6 inhibition resulted in high sensitivity and resensitized tumors to CDK4/6 inhibition^118,119^. As such, *CCNE1* amplification in resistant tumors may also serve as a biomarker of response to combined CDK2 and CDK4/6 inhibition.

## The contribution of the tumor microenvironment

Recent studies have revealed that CDK4/6 inhibitors not only halt cancer cell proliferation but also increase anti-tumor immunity. For example, abemaciclib treatment significantly increased antigen presentation on MHC class I molecules^120^. Mechanistically, CDK4/6 inhibition enhances antigen presentation by suppressing the Rb–E2F axis and downregulating the expression of DNMT1, an E2F target. This leads to the demethylation of endogenous retroviral elements, resulting in the production of type III interferons and the upregulation of MHC class I molecules. In addition, CDK4/6 inhibition has been reported to increase MHC class II molecules and induce new antigen ligands on HR^+^ breast cancer cells, further improving immune visibility^121,122^.

Several studies have indicated that CDK4/6 inhibition augments T cell activation by reducing the proliferative capacity of immunosuppressive regulatory T (T_reg_) cells^120,123,124^. T_reg_ cells are known to suppress immune responses, promote tumor immune evasion and are associated with poor prognosis in patients with breast cancer^125^. Notably, T_reg_ cells express higher levels of CDK6 than other T cell subtypes, possibly increasing their sensitivity to CDK4/6 inhibition^124^. In mouse models treated with abemaciclib, the number of proliferating T_reg_ cells significantly decreased, whereas CD8^+^ T cell levels remained unchanged, reducing the T_reg_-to-CD8^+^ T cell ratio^120^. Clinical studies have corroborated these findings, revealing significant downregulation of circulating T_reg_ cells in HR^+^/HER2^−^ breast cancer during CDK4/6 inhibitor treatment^123^. This reduction in T_reg_ cells is correlated with an improved treatment response, suggesting that altering the T_reg_-to-CD8^+^ T cell ratio may alleviate immune suppression and enhance CD8^+^ T cell function.

Other studies have indicated that CDK4/6 inhibition augments the transcriptional activity of the NFAT family of transcription factors in activated T cells^121,124^. NFAT proteins are essential for T cell activation and regulate key cytokines such as IL-2, IL-4 and IFNγ^126–129^. Palbociclib treatment has been shown to increase NFAT nuclear localization and transcriptional activity in T cells, leading to elevated expression of NFAT target genes essential for T cell activation and anti-tumor immunity^124^. Similarly, abemaciclib treatment sustained NFAT nuclear retention, prolonging T cell activation by delaying NFAT phosphorylation^121^.

Modulation of the immunosuppressive factor PD-L1 by CDK4/6 inhibitors has also been observed, although this relationship is complex and context dependent. In one study, CDK4 was shown to phosphorylate and stabilize the SPOP E3 ligase, facilitating the ubiquitination and degradation of PD-L1^130^. CDK4/6 inhibition disrupts this process, resulting in PD-L1 accumulation on the cell surface in TNBC and HR^+^/HER2^−^ tumors, which may contribute to an immunosuppressive tumor microenvironment by promoting immune evasion. However, other studies reported that palbociclib treatment reduces PD-L1 expression by repressing E2F-mediated transcription^131^. These contrasting findings suggest that the effect of CDK4/6 inhibitors on PD-L1 expression depends on the tumor context, the signaling pathways involved, or the CDK4/6 inhibitor used.

Despite these promising preclinical findings, translating these immunomodulatory effects of CDK4/6 inhibition into clinical practice has been challenging due to hematopoietic toxicity. In phase I and II trials, hematopoietic toxicity has been the primary dose-limiting toxicity of FDA-approved CDK4/6 inhibitors, necessitating intermittent drug treatment^60,132–134^. One study reported grade 3 hematologic toxicity, including neutropenia, anemia and leukopenia, occurring as early as the first treatment cycle, which involved 3 weeks of dosing followed by a 1-week break^132^. Preclinical and clinical studies have shown that CDK4/6 inhibitors impair bone marrow function, reducing the production of immune cells essential for effective tumor clearance^112,132,135^. Hematopoietic cells, including bone marrow progenitors, rely heavily on CDK4/6 for proliferation, making them particularly susceptible to these inhibitors^135^. In addition, CDK4/6 inhibition decreases the number of myeloid cells, which are crucial for antigen presentation and immune response coordination within the tumor microenvironment^124,136^. This reduction in immune cell proliferation complicates the balance between enhancing anti-tumor immunity and managing the toxic effects of CDK4/6 inhibitors.

In conclusion, the suppression of bone marrow function and associated reduction in immune cell production underscore the challenges of translating the preclinical immunomodulatory benefits of CDK4/6 inhibitors into clinical success. Continued research into the underlying mechanisms will be vital for optimizing the therapeutic potential of CDK4/6 inhibitors, especially in combination with other immunotherapies, while minimizing adverse effects.

## Potential therapeutic strategies to overcome CDK4/6 inhibitor resistance

Despite the inevitable development of resistance to CDK4/6 inhibitors, no consensus has been established for second-line treatment options after disease progression. Several retrospective studies have provided valuable insights into potential treatment strategies. Multiinstitutional patient database analyses suggest that patients who continue with targeted therapies after progressing on CDK4/6 inhibitors have significantly improved PFS compared with those who switch to chemotherapy^137–140^. This trend underscores the importance of prioritizing continued targeted therapy as a second-line approach rather than shifting to systemic chemotherapy.

### PI3K/AKT/mTOR inhibitors combined with endocrine therapy

Mutations and activation of the PI3K/AKT/mTOR pathway are common among patients with HR^+^/HER2^−^ breast cancer^141,142^, making inhibitors of this pathway a logical choice after progression. Clinical trials of PI3K inhibitors combined with endocrine therapy have shown benefits in patients with *PIK3CA* mutations following progression on CDK4/6 inhibitors^143,144^. The CAPItello-291 trial evaluated AKT inhibitors in patients who had progressed on endocrine therapy (with or without CDK4/6 inhibitors), revealing a significant PFS benefit, particularly in patients with *PIK3CA*/*AKT1*/*PTEN* alterations^145^. This leads to the approval of the AKT inhibitor capivasertib for such mutations. Another ongoing trial is investigating the efficacy of the other AKT inhibitor ipatasertib in similar settings (NCT04650581). In addition, the use of mTOR inhibitors combined with endocrine therapy has demonstrated promise after progression with CDK4/6 inhibitors^146,147^.

### Maintenance of CDK4/6 inhibitors with endocrine therapy

Several trials have investigated the potential benefits of continuing CDK4/6 inhibitors beyond disease progression. The PACE and PALMIRA trials assessed palbociclib continuation with a new endocrine therapy following resistance to CDK4/6 inhibitors, but these studies reported no significant improvement in PFS^148,149^. However, the MAINTAIN and postMONARCH trials, which examined second-line treatments using ribociclib and abemaciclib, demonstrated enhanced patient responses, suggesting that switching to a different CDK4/6 inhibitor may be beneficial after progression on palbociclib^150–152^. Retrospective analyses further support this maintenance approach, with higher response rates observed in patients treated with abemaciclib-based regimens^153^. A recent preclinical study indicated that continued CDK4/6 inhibition in drug-resistant cells activates a noncanonical pathway, leading to Rb degradation rather than phosphorylation for inactivation^88^. This mechanism results in low and gradual E2F activation kinetics, significantly attenuating drug-resistant tumor growth. Notably, this reduced E2F activity slows cell cycle progression without affecting cell cycle entry, suggesting that OS, rather than PFS, is probably a more suitable primary endpoint to capture the full benefit of sustained CDK4/6 inhibitor therapy.

### Targeting CDK2, with or without CDK4/6 inhibitors

While CDK1 knockout in mice results in embryonic lethality, mice develop normally with either CDK4/6 or CDK2 knockout^41,154,155^, suggesting that neither CDK4/6 nor CDK2 is essential for cell proliferation. Further research using mice lacking CDK2/3/4/6 revealed that CDK1 alone is sufficient to drive cell cycle progression^156^. Given the critical role of CDK2 activity in the development of resistance to CDK4/6 inhibitors^118,119,157,158^, several studies have highlighted the potential of dual targeting of CDK4/6 and CDK2 activities to overcome resistance^88,118,119,157,158^. Despite this therapeutic promise, the clinical development of CDK2/4/6 inhibitors has been hampered by their prohibitive toxicity^159,160^. However, interim results from another trial suggested that combining CDK2 and CDK4/6 inhibitors may be feasible with manageable toxicity^161^. Ongoing clinical trials, including NCT05252416 and NCT05735080, are assessing the efficacy of CDK2 inhibitors, such as INX-315 and BLU-222, as monotherapies and in combination with endocrine therapies, with or without CDK4/6 inhibitors.

### CDK7 inhibitor combined with endocrine therapy

While the effects of CDK7 inhibitors on breast cancer are not yet fully understood, CDK7 is known to play a dual role in cell cycle progression and transcription initiation by phosphorylating RNA polymerase II and priming cell cycle CDKs for activation, making it an attractive therapeutic target^162^. Selective CDK7 inhibitors have demonstrated greater tumor sensitivity than normal cells, driving apoptosis in CDK4/6 inhibitor-resistant breast cancer models^163,164^. A phase I clinical trial of the CDK7 inhibitor samuraciclib with endocrine therapy has shown encouraging clinical activity, suggesting that CDK7 inhibition is a potential approach for resistant breast cancers^165^. Other trials will provide insights into the safety and efficacy of CDK7 inhibitors after progression on CDK4/6 inhibitors^166^.

### Immunotherapy

Preclinical studies suggest that CDK4/6 inhibitors can inhibit T_reg_ cell proliferation and upregulate MHC molecule expression on tumor cells^120–124^, potentially enhancing anti-tumor immunity in breast cancer. However, initial clinical trials combining immune checkpoint inhibitors (ICIs) with CDK4/6 inhibitors in metastatic breast cancer did not yield significant PFS improvements^167–169^. In addition, severe adverse effects, including hepatotoxicity, led to treatment discontinuations. In the NEWFLAME trial, a combination of nivolumab (anti-PD1), abemaciclib and endocrine therapy demonstrated anti-tumor activity in HR^+^/HER2^−^ metastatic breast cancer^170^. Although efficacy was promising, the trial was halted because of severe immune-related adverse events. Conversely, the PACE trial reported a significant increase in PFS with a combination of palbociclib, endocrine therapy and avelumab (anti-PD-L1) compared with palbociclib and endocrine therapy alone in patients previously treated with CDK4/6 inhibitors^171^. Additional trials are examining the safety and efficacy of combining CDK4/6 inhibitors with ICIs at various stages of breast cancer (NCT04841148 and NCT03280563). Given the overlapping toxicities of ICIs and CDK4/6 inhibitors, further research is essential to optimize this strategy.

## Conclusions and future perspectives

Targeting the cell cycle machinery in breast cancer is a promising strategy for enhancing therapeutic outcomes. While CDK4/6 inhibitors have reshaped the treatment landscape, addressing resistance and exploring novel targets and therapeutic approaches are crucial for sustaining their effectiveness. Future research focused on combination therapies, noncanonical cell cycle entry pathways and the tumor microenvironment could pave the way for more effective and lasting treatments. Integrating these strategies into clinical practice will ultimately improve the management and prognosis of patients with breast cancer.
